# Individual Creativity in Digital Transformation Enterprises: Knowledge and Ability, Which Is More Important?

**DOI:** 10.3389/fpsyg.2021.734941

**Published:** 2022-01-12

**Authors:** Daokui Jiang, Zhuo Chen, Teng Liu, Honghong Zhu, Su Wang, Qian Chen

**Affiliations:** ^1^Business School, Shandong Normal University, Jinan, China; ^2^School of Innovation and Entrepreneurship, Shandong University, Qingdao, China; ^3^School of Economics, Ocean University of China, Qingdao, China

**Keywords:** digital transformation, person-organization fit, work engagement, individual creativity, polynomial regression analyses

## Abstract

Digital technological innovation is reshaping the pattern of industrial development. Due to the shortage of digital talents and the frequent mobility of these people, the competition for talents will be very fierce for organizations to realize digital transformation. The digitization transformation of China’s service industry is far ahead of that of industry and agriculture. It is of great significance to study the organizational management and talent management of service enterprises to reduce the negative impact of insufficient talent reserve and meet the needs of digital development. Based on 378 valid questionnaires from China’s service industry, this paper applied polynomial regression and a response surface model to analyze the impact of two kinds of person-environment fit on work engagement and individual creativity. The results show that: (1) under the combination of high morality and high talent, work engagement and individual creativity are the highest; (2) individual creativity is stronger under the condition of high morality and low talent than under low morality and high talent; and (3) work engagement mediates the influence of morality and talent on individual creativity. The research reveals the internal mechanism by which morality and talent cooperatively promote individual creativity, which provides theoretical guidance for management practice of service firms to improve individual creativity in the process of digital transformation.

## Introduction

With the development of informatization, networking, digitization, and intelligence, the world is entering an era characterized by digital productivity. Digital technological innovation has accelerated the transformation of economic and social forms and operation modes. A broader and deeper scientific and technological revolution and industrial transformation are reshaping the global landscape of innovation and the pattern of industrial development (State Information Center [SIC] and Jingdong Digital Technology Research Institute [JDTRI], 2020). The digital transformation of China’s service sector is far ahead of manufacturing industry and agriculture, which is benefited from the strong domestic consumer market and the demand for digital transformation of the service sector. According to China Academy of Information and Communications Technology [CAICT], 2019), digital economy, respectively, accounts for 18.3, 35.9, and 7.3% of added value in China’s industry, service industry and agriculture in 2018. And a number of Chinese service companies such as BATJ (Baidu, Alibaba, Tencent, Jingdong), TMDP (Toutiao, Meituan, Didi, Pinduoduo) have entered the top 20 Internet companies in terms of market capitalization. The application of digital technology to the service sector will cultivate new forms of business such as smart logistics, e-commerce and smart finance, and accelerate the high-quality and efficient development of producer and consumer services. However, 30% of technology jobs will be vacant by 2020 due to a shortage of digital talents, which means the competition for talents in the digital transformation of enterprises will be fierce (State Information Center [SIC] and Jingdong Digital Technology Research Institute [JDTRI], 2020). Knowledgeable talents have a strong willingness to flow (Furusawa and Brewster, 2015) and the turnover of this type of talents is relatively frequent. Especially in information technology (IT) industry, the flow of talents is more frequent than that of other industries. Therefore, it is of great significance to study the organization and management of China’s service industry enterprises and explore the impact of human-environment matching on employees’ work input and creativity, so as to adjust the human resource structure of digitalized transformation enterprises and ensure that digital technology professionals can meet the technical needs of digitalized transformation enterprises. The importance of morality and talent for organizations has always been a controversial topic, especially in China, which is deeply influenced by traditional culture and considers morality to be most important. Influenced by realism, talent has received more attention in theory and practice. In terms of personnel selection, training, inspiration, and retention, organizations pay far more attention to the talents of employees than other aspects. In contrast, morality is not valued as much as it should be. The person-environment fit theory provides a good perspective for studying how individual behaviors contribute to the achievement of organizational goals and has also received much attention. However, research on person-environment fit theory mainly focuses on the fields of health and stress (Edwards and Cooper, 1990), job adaptation, career choice, and organizational culture (Schneider, 1987). In an era of digital economy, in which creativity is becoming increasingly important, the influence mechanism of person-environment fit on individual creativity is important. The theory of person-environment fit provides support for the study of individual creativity. Matching theory (Schneider, 1987) suggests that matching can predict individual outcomes (such as job satisfaction) better than any single component (person or environment). The match between individuals and the environment is a complex, dynamic process (Milliman et al., 2017). Individuals are easily attracted by organizations whose values and goals are similar to their own (Schneider, 1987). The higher the degree of matching between individuals and the environment, the greater individuals’ satisfaction and job performance will be, and the lower their work pressure and turnover intention will be. However, research on the impact of values on creativity is insufficient.

Creativity is becoming a core competency for employees, and most modern organizations encourage creativity. Individual creativity is the result of the interaction between individual characteristics and environmental factors. Individual characteristics include intrinsic motivation (Hannam and Narayan, 2015), cognitive style (Wu et al., 2014), goal orientation (Miron-Spektor and Beenen, 2015), and self-efficacy. Environmental factors include organizational culture, group size, task characteristics, and work pressure (Baer and Oldham, 2006). Individual creativity is an important source for organizations to innovate and maintain competitive advantages (Woodman et al., 1993).

Work engagement has become an important topic in the field of human resource management (Alzyoud, 2018) and has an important impact on the competitive advantage of organizations. Employees with a high level of work engagement are physically, cognitively, and emotionally attached to the organization and engaged in work with great enthusiasm. Work engagement has a direct impact on employee productivity, resulting in higher customer satisfaction, higher productivity, and a lower turnover rate, which ultimately translates into the improvement of overall performance (Maslach et al., 2001). Training and using highly dedicated employees is an important means for enterprises to gain competitive advantage.

Based on matching theory, this study applies the response surface regression method to explore how morality and talent affect individual creativity by affecting work engagement. This study compares the impact of different matches of morality and talent on work engagement and individual creativity to provide a reference for service industry organizations in the context of digital transformation to better stimulate individual creativity. The contribution of this study is mainly reflected in the following aspects. First, combined with person-environment fit theory, this study enriches research on the antecedents of individual creativity and reveals the impact of person-environment fit on individual creativity. Second, the paper compares and analyzes the influence of different types of person-environment fit on individual creativity. In this study, the effects of different states of morality and talent on work engagement and individual creativity are compared to better understand the influence mechanism of the interaction between person-environment fit and Individual Creativity. Third, this study explores the mechanism by which morality and talent fit with individual creativity and reveals the mediating role of work engagement in this process. This study provides a reference for further understanding of the relationship between person-environment fit, work engagement and individual creativity.

This paper began with an introduction to the overarching theory that guided the studies and contributed to the formulation of the hypotheses to be tested. Subsequently, we describe the overview of the analysis strategy, data collection, variable measures, and data analysis to test the hypotheses. Finally, this paper is concluded with a discussion of the results, implications, limitations, and future research avenues.

## Literature Review and Hypothesis

### Person-Environment Fit Theory

Matching theory suggests that individuals have a natural need to adapt to the environment and to find an environment that suits their characteristics. Individuals hope to exercise control over their lives, reduce uncertainty, and increase their sense of belonging. For matching theory, relevant areas of study include person-job fit, person-subordinate fit, person-team fit, person-organization fit, and person-environment fit. Among these, person-job fit includes two dimensions, demand-ability fit and need-supply fit (Cable and DeRue, 2002); person-subordinate fit and person-team fit mainly focus on the consistency of values between individuals and their colleagues; and person-organization fit, or consistency fit considers individuals and organizations in achieving the goal of the consistency of values. Person-environment fit includes person-job fit (complementary fit) and person-organization fit (consistency fit) (Kristof-Brown, 2000; Kristof, 2006); among these, consistency fit is the most important factor affecting person-environment fit (Rounds et al., 1987; O’Reilly et al., 1991; Vecchione et al., 2016).

Research on matching theory shows that matched relationships have positive effects on employees’ mental health (Caplan, 1987; Hogg, 2000; Deci and Ryan, 2008), personal willingness (Chatman, 1989; Vansteenkiste et al., 2007; Shen et al., 2018), job satisfaction (Gregory et al., 2010), organizational commitment (Milliman et al., 2017), job performance and organizational citizenship behavior (Nye et al., 2012; Marstand et al., 2017), and interpersonal relationships (Hogg, 2000; Edwards and Cable, 2009). In contrast, mismatched relationships have a negative impact on counterproductive work behavior (Van Iddekinge et al., 2011; Nye et al., 2017). It has also been found that matching has the most significant effect on basic outcomes (work attitude, such as satisfaction) (Judge et al., 2002; Steel et al., 2008) but does not have a significant effect on behavioral outcomes (such as performance, turnover rate, and work choice) (Judge and Bono, 2001; Bakker et al., 2014). The weak impact of consistent fit on performance and turnover rate often occurs through work attitude (Arthur et al., 2006).

In reality, however, differences tend to inspire individuals to change (Bandura, 1991; Longua et al., 2009). Therefore, optimal fit rarely exists, and perfect fit may even impair an individual’s ability to learn, develop, and adapt. When both personal and environmental attributes are high, optimal results will appear (Slocombe and Bluedorn, 1999; Hecht and Allen, 2005). Studies have shown that changes in individuals’ attitudes and behaviors are the result of interactions between individuals and the environment, which cannot be explained by individuals or the environment alone (Edwards, 1996). In previous studies on the impact of fit on results, half of the studies have found a nonlinear relationship between the impact of person-organization fit on results (Lambert et al., 2012; Marstand et al., 2017). These nonlinear relationships mainly depend on the specific attributes of person-organization fit (Crawford et al., 2010; Newton and Mazur, 2016).

### The Influence of Morality and Talent Fit on Individual Creativity

Values are a unique way of thinking and a code of conduct formed by an individual, and their influence on individual psychology and behavior is long term and fundamental. The basic beliefs shared by all members of an organization constitute organizational values (O’Reilly et al., 1991). Research shows that the consistency of personal and organizational values has a promoting effect on job satisfaction (Verplanken, 2004; Edwards and Cable, 2009). Choi (2004) found that the consistency of values and demand-ability fit have a positive impact on individual innovation behavior. In terms of traditional Chinese culture, the consistency of values can be called morality, and demand-ability fit can be called talent. Managers hope that the members of the organization have a high level of morality and talent. In a state of high morality and talent, that is, consistency of values and demand-ability fit, individuals are more likely to accurately position their roles and engage in more extra-role behaviors (Yaniv et al., 2010). The consistency of values between individuals and organizations enables individuals to perceive a stronger organizational atmosphere and generate positive emotions, which have a positive impact on the formation of creativity. Demand-ability fit means that an individual’s professional skills, knowledge and experience are in line with the requirements of the job (Kristof-Brown, 2000). Individuals feel confident and enthusiastic in their work, which generates positive behaviors (Chatman, 1991; Resick et al., 2007). Therefore, an individual with demand-ability fit has more solid knowledge of the field, a higher sense of creative self-efficacy, and a stronger influence on creativity. Consistency of values and demand-ability fit leads to a more creative organizational atmosphere, more solid professional knowledge, more positive emotions, and more powerful creative motivation and helps employees to be more creative.

Consistency of values is different. Low consistency of values indicates the lack of a correct evaluation of one’s role and weak identification with organizational goals and is likely to generate negative emotions (Vandenberghe, 1999), all of which are not conducive to the formation of individual creativity. When demand-ability fit is low, ability exceeds job requirements, or the individual’s ability fails to meet the job requirements. Vianen (2018) proved in his research that when individual characteristics are consistent with environmental characteristics, the optimal output can be obtained, which does not have a significant relationship with the individual absolute level. In view of this, individuals who have consistency of values and demand-ability fit with an organization are more likely to show positive work behaviors, which are conducive to the formation of individual role cognition and creative motivation. Individuals with high talent are more likely to break through the shackles of inherent patterns, enhance their creative self-efficacy, and make efforts to improve organizational creativity. In other words, when demand-ability fit is high, individuals will be more comfortable with their work, generate creative motivation, and use their professional knowledge and skills to improve creativity.

With regard to the mismatch between morality and talent when the degree of consistency of values is high and the degree of demand-ability fit is low, the degree of consistency of values means that individuals have high recognition of organizational goals and norms, and these individuals are likely to achieve better relationship performance (Kristof-Brown, 2000). When the degree of demand-ability fit is low, individuals can improve their abilities to meet job requirements or change their cognition of job requirements to improve the degree of demand-ability fit. When the degree of consistency of values is low and the degree of demand-ability fit is high, it is difficult for individuals to obtain a sense of belonging and support in the organization, which is not conducive to the formation of a creative atmosphere. Based on the above analysis, the following hypotheses are proposed:

*Hypothesis 1a:* Compared with employees with a morality and talent misfit, employees are more creative when there is a fit between morality and talent.

*Hypothesis 1b:* Under the condition of morality and talent fit, compared with the state of low morality and low talent level, employees with high morality and high talent level are more creative.

*Hypothesis 1c*: Under the condition of morality and talent misfit, compared with the state of low morality and high talent level, employees with high morality and low talent level are more creative.

### The Influence of Morality and Talent Fit on Work Engagement

Under the condition of morality and talent fit, individuals with high consistency of values are more likely to form higher organizational identity and a sense of belonging, be clearer about their positioning in the organization, and have more positive work behaviors (Yaniv et al., 2010). When demand-ability fit is high, individuals are more skilled in coping with work and experience a sense of accomplishment and control in work. When an individual’s ability meets work requirements, he or she will have higher internal work motivation and work involvement (Deci and Ryan, 2008). When there is a misfit between morality and talent, individuals and organizations have low consistency of values, which will lead to higher turnover intention and difficulty in achieving work engagement. A low level of demand-ability fit causes the self-efficacy of individuals in the organization to be low, which affects individual behavior motivation (Bandura and Locke, 2003). Compared with morality and talent misfit, an individual’s work engagement is higher in the condition of morality and talent fit.

When there is a morality and talent fit, the degree of consistency of values and demand-ability fit is high. Individuals can experience more enjoyment in their work and will have higher motivation for independent work and a higher level of work engagement. When morality is low and talent is low, individuals’ work engagement is mostly related to organizational incentives or punishments, and they passively engage in work. From this point of view, when morality and talent fit, individuals’ work engagement is higher when the degree of consistency of values is high and talent is high.

When morality is high and talent is low, due to the identification with organizational values, individuals have a strong sense of their membership, and they will make efforts to align themselves with the needs of the organization and increase their work input. However, it is difficult to feel a sense of support and security in the organization when morality is low and talent is high. Even if individuals’ ability meets the needs of the organization, it is difficult to change their values, which is not conducive to the increase of individual work input. From this point of view, when there is a morality and talent misfit, individuals’ work engagement is higher when morality is high and talent is low. Based on the above analysis, the following hypotheses are proposed:

*Hypothesis 2a:* Compared with a morality and talent misfit, an individual’s work engagement is higher when there is a morality and talent fit.

*Hypothesis 2b:* Under the condition of a morality and talent fit, compared with the state of low morality level and low talent level, an individual’s work engagement is higher under the condition of a high morality level and a high talent level.

*Hypothesis 2c:* Under the condition of morality and talent misfit, compared with the state of a low moral level and a high talent level, an individual’s work engagement is higher under the condition of a high moral level and a low talent level.

### The Mediating Role of Work Engagement

According to person-environment fit theory, person-environment fit can have a positive impact on individual attitudes and behaviors (Chatman, 1989). For both organizations and individuals, values play a guiding role in behavior. When there is a high degree of consistency between organizational and individual values, there is a close emotional connection between the individual and the organization. Individuals’ identification with their own organizational identity encourages them to increase their work engagement and perform more extra-role behaviors. When there is a high level of demand-ability fit, individuals can skillfully deal with problems in work and obtain a high sense of self-efficacy in practice. The higher an individual’s self-efficacy, the higher his or her work engagement (Bandura, 1988). Studies have shown that the effects of different matching patterns on individual attitudes and behaviors are not simply additive, and the interaction between them can also affect individual behavior (Kristof-Brown, 2000).

Work engagement describes the mental state of an individual who is engaged in work (Hallberg and Schaufeli, 2006). With a high morality and talent fit, individuals can successfully solve difficulties at work by virtue of their professional knowledge and skills, meet their need for autonomy, competence and relationship in work, and increase work input. Studies have shown that work engagement has positive effects on organizational citizenship behavior (Kataria et al., 2013), job satisfaction (Schaufeli et al., 2006), and low turnover intention (Schaufeli and Bakker, 2004). Individuals with high work engagement are consistently enthusiastic at work and are willing to devote time and energy to things other than work. Individuals with a high morality and talent fit have higher work input. They can use their subjective initiative in the face of difficulties in work, combine existing professional knowledge, cultivate creative thinking, and improve individual creativity. Based on the above analysis, the following hypothesis is proposed:

*Hypothesis 3:* Work engagement mediates the relationship between morality and talent fit and individual creativity.

## Materials and Methods

### Data Collection

In this study, a questionnaire survey was used to obtain research data. The link to the designed questionnaire was sent to front-line service workers in enterprises that are in digital transformation to complete. To reduce common method variance, the following measures were taken: (1) the questionnaire was translated into Chinese, and then three professionals were asked to translate it into English to ensure the accuracy of the information; (2) the questionnaires had been tested before they were sent out; (3) the questions were randomly ordered to reduce homologous error; and (4) data samples with short answer time and incomplete data were deleted. A total of 500 questionnaires were sent out and 398 were recovered, with a recovery rate of 79.6%. After eliminating invalid samples, a total of 378 valid questionnaires were received, with an effective rate of 95.0%. According to the results of the statistical analysis, in terms of gender, there were 173 men, accounting for 45.8%, and 205 women, accounting for 54.2%. In terms of age, 71 respondents were under 25, accounting for 18.8%, 228 respondents were 26–35 years old, accounting for 60.3%, 72 respondents were 36–45 years old, accounting for 19.0%, and 7 respondents were over 46 years old, accounting for 1.9%. In terms of education level, 150 respondents had a junior college education, accounting for 39.7%, 141 had a bachelor’s degree, accounting for 37.3%, and 87 had a graduate degree, accounting for 23.0%.

### Measures

The survey measures included morality, talent, work engagement and individual creativity. All of the measurement scales were well established and drawn from the literature. The survey was administered in Chinese. All items used the same five-point Likert scale format (1 = strongly disagree; 5 = strongly agree).

#### Morality

Morality was measured by the persistence of values using a 6-item scale developed by Cable and DeRue (2002). Example items included “I strongly agree with the organization’s goals” and “My values are similar to the organization’s values.” The internal consistency Cronbach’s α was 0.951.

#### Talent

Talent was measured by demand-ability fit using a 3-item scale developed by Cable and DeRue (2002). Example items included “My abilities and training are well matched with the job requirements” and “My personal abilities and education level are well matched with the job requirements.” The internal consistency Cronbach’s α was 0.921.

#### Work Engagement

Work engagement was measured using a 17-item scale developed by Schaufeli et al. (2006) using a simplified version with 9 questions. Example items included “I am passionate about my work” and “I am proud of what I do.” The internal consistency Cronbach’s α was 0.961.

#### Individual Creativity

Individual creativity was measured using a 7-item scale developed by Zhou and George (2011). Example items included “I will sell my new ideas and ideas to other colleagues” and “I will strive for the resources needed to realize the new ideas.” The internal consistency Cronbach’s α was 0.958.

Correlation coefficient matrix is shown in Table 1.

**TABLE 1:** Correlation coefficient matrix.

	Mean value	SD	1	2	3	4	5	6	7
1. Gender	1.540	0.499	1						
2. Age	2.040	0.672	-0.080	1					
3. Education level	1.830	0.775	0.097	0.186**	1				
4. Talent	3.836	0.814	-0.013	-0.004	-0.129*	***0.864***			
5. Morality	3.867	0.849	0.042	-0.033	-0.197**	0.657**	***0.838***		
6. Work engagement	3.938	0.815	0.031	0.011	-0.243**	0.678**	0.787**	***0.771***	
7. Individual creativity	3.920	0.715	-0.077	0.015	-0.069	0.551**	0.606**	0.652**	***0.801***

* and **, respectively, indicate significance at the level of p < 0.05 and p < 0.01.

*The bold values are the square root of average variance extracted (AVE).*

### Data Analysis Method

In this study, polynomial regression and response surface methods were used to test the hypotheses. This method is suitable for testing the degree of correlation between two predictive variables and their mutual consistency, difference and outcome variables (Edwards, 1994; Edwards et al., 2006). In recent years, it has been widely valued and applied (Harris et al., 2014; Shanock et al., 2014; Lee et al., 2016; Bar-Kalifa et al., 2017; Weidmann et al., 2017; Yang et al., 2017; Audenaert et al., 2018; Chen et al., 2019; Qiu et al., 2020; Jiang et al., 2021).


(1) $$Theregressionmodelis:Z=b_{0}+b_{1}X+b_{2}Y+b_{3}X^{2}+b_{4}\,X\,Y+b_{5}\,Y^{2}+\varepsilon$$

It can be seen from Eq. (1) that the regression equation needs to calculate the regression coefficients of X and Y, X2, and Y2, and XY. Before analysis, the independent variables X and Y need to be centralized to reduce multicollinearity (Edwards and Parry, 1993).

Response surface technology has three key indicators: fixed point, principal axis, slope and curvature. (1) The principal axis describes the direction of the response surface on the X-Y axis. The first and second principal axes are perpendicular to each other and intersect at a fixed point. The shape of the response surface can be judged according to the spindle. For a convex surface, the curvature will be greatest along the first principal axis, and the second principal axis will have the least curvature. For a concave surface, the curvature downwards along the first principal axis is the smallest, while the curvature along the second principal axis is the largest. (2) With regard to slope and curvature, the consistency line refers to two measurement index values that are equal and in the same direction on the X-Y plane (X = Y). An incongruent line is a line in the X-Y plane where two measurement indicators are equal but opposite in direction (X = -Y). By substituting X = Y and X = -Y into Eq. (1), the consistency line and inconsistency line are calculated as follows:


(2) $$Z=b_{0}+\left(b_{1}+b_{2}\right)X+\left(b_{3}+b_{4}+b_{5}\right)X^{2}+\varepsilon$$

(3) $$Z=b_{0}+\left(b_{1}-b_{2}\right)X+\left(b_{3}-b_{4}+b_{5}\right)X^{2}+\varepsilon .$$

Along the line Y = X, the slope is (b1 + b2) and the curvature is (b3 + b4 + b5). Along the line Y = -X, the slope is (b1-b2) and the curvature is (b3-b4 + b5). When (b3 + b4 + b5), (b3-b4 + b5) is positive and significant, which means that the line is concave (U-shaped); on the contrary, when the value is negative, it is convex (inverted U-shaped).

## Results

The analysis results are shown in Table 2. First, taking work engagement as the dependent variable, the results of model 1 show the main effects of the control variables. Morality and talent have a significant positive impact on work engagement, but the effect of talent on work engagement is lower than that of morality (β = 0.283 < β = 0.558).

**TABLE 2:** Polynomial regression results.

Variables	Work engagement		Individual creativity		
	Model 1	Model 2	Model 3	Model 4	Model 5
Constant term	3.945***	3.963***	4.007***	3.909***	2.212***
Gender	0.040	0.046	-0.140*	-0.140*	-0.159**
Age	0.064	0.062	0.013	0.018	-0.008
Education level	-0.110**	-0.115***	0.056	0.073	0.122***
Morality, β1	0.558***	0.576***	0.379***	0.415***	0.168**
Talent, β2	0.283***	0.255***	0.229***	0.247***	0.138**
Morality^2^, β3		0.096**		-0.015	-0.057
Morality × Talent, β4		-0.139**		0.118	0.177**
Talent^2^, β5		-0.029		0.019	0.032
Work engagement					0.428***
Slope: β1 + β2	-	0.781***	-	0.662***	0.306***
Curvature: β3 + β4 + β5	-	-0.072*	-	0.121***	0.152***
Slope: β1-β2	-	0.321***	-	0.168	0.030
Curvature: β3-β4 + β5	-	0.026**	-	-0.114	-0.202*
△*R*^2^	-	0.013**	-	0.025**	0.074***
*F*	154.794	101.980	53.964	36.981	44.154

*, **, and ***, respectively, indicate significance at the level of p < 0.05, p < 0.01, and p < 0.001.

Model 2 shows that the influence of morality and talent on work engagement is significant on the consistency line (β1 + β2 = 0.781, *p* < 0.001) and curvature (β3 + β4 + β5 = −0.072, *p* < 0.05); that is, when morality and talent are the same, the impact of the two on work engagement is in an inverted U shape, and the size needs to be re-examined. On the inconsistency line, the slope is significant (β1-β2 = 0.321, *p* < 0.001) and the curvature is significant (β3-β4 + β5 = 0.026, *p* < 0.05); that is, when morality and ability are inconsistent, the impact of the two on work engagement is U-shaped, and the size needs to be re-examined.

Taking individual creativity as the dependent variable, the results of Model 3 show the main effect of the control variables. Morality and talent have a significant positive impact on individual creativity, but the effect of talent on work engagement is lower than that of morality (β = 0.229 < β = 0.379). The results of Model 4 show that the influence of morality and talent on individual creativity is significant on the consistency line (β1 + β2 = 0.662, *p* < 0.001) and the curvature is significant (β3 + β4 + β5 = 0.121, *p* < 0.001); that is, when morality and talent are the same, the impact of the two on individual creativity is U-shaped, and the size needs to be re-examined. On the inconsistency line, the slope is not significant (β1-β2 = 0.168, *p* > 0.05) and the curvature is not significant (β3-β4 + β5 = −0.114, *p* > 0.05); that is, when morality and talent are inconsistent, their impact on individual creativity is flat.

**FIGURE 1:**
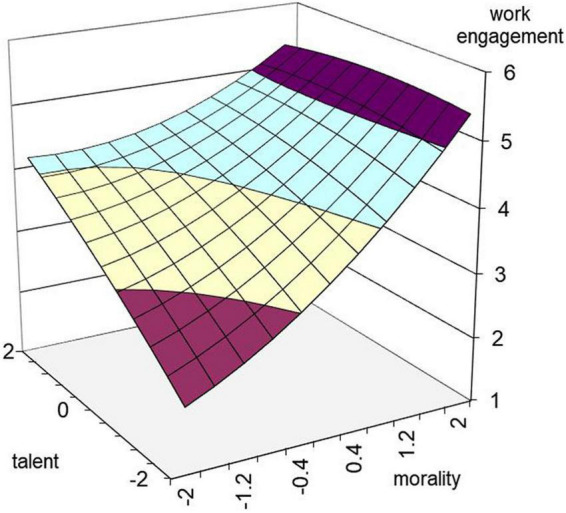
Effects of morality and talent on work engagement.

**FIGURE 2:**
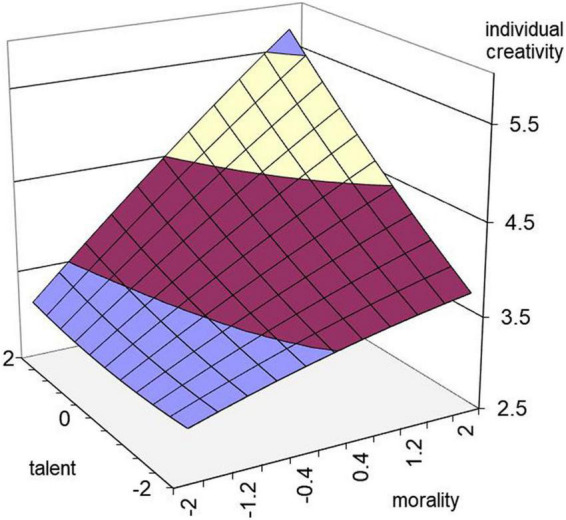
Effects of morality and talent on individual creativity.

To analyze the comprehensive influence of talent and morality on work engagement and individual creativity, a two-way ANOVA was conducted with morality and talent as independent variables and work engagement and individual creativity as dependent variables. The results show that in the model with work engagement as the dependent variable, the interaction between talent and morality is significant, *F* = 101.670, *p* < 0.001, *R*^2^ = 0.658 (Figure 1). In the model with individual creativity as the dependent variable, *F* = 58.75, *p* < 0.001, *R*^2^ = 0.525 (Figure 2). According to relevant scholars, the two groups were divided into high and low morality and high and low talent according to the standard of 27%. Taking morality (high and low groups) and talent (high and low groups) as independent variables and taking work engagement and individual creativity as dependent variables, the simple effect was further analyzed, and the results are shown in Table 3. Through mean comparison, it can be seen that high morality-high talent > high morality-low talent > low morality-high talent > low morality-low talent, which means that the combination of high morality and high talent can promote the transformation process of work engagement and individual creativity. These results support Hypotheses 1a, 1b, 1c, 2a, 2b, and 2c.

**TABLE 3:** Interaction effect analysis of morality and talent (*N* = 158).

Dependent variable	Types	Mean	SD	95% Confidence interval		Comparison
				Lower limit	Upper limit	
Work engagement	High M-High T	4.738	0.074	4.593	4.883	High M-High T> High M-Low T> Low M-High T> Low M-Low T
	High M-Low T	4.723	0.318	4.093	5.352	
	Low M-High T	3.776	0.241	3.3	4.251	
	Low M-Low T	2.926	0.075	2.777	3.074	
Individual creativity	High M-High T	4.68	0.074	4.534	4.826	High M-High T> High M-Low T> Low M-High T> Low M-Low T
	High M-Low T	3.965	0.32	3.334	4.596	
	Low M-High T	3.694	0.242	3.217	4.172	
	Low M-Low T	3.286	0.075	3.137	3.435	

*M, morality; T, talent.*

Bootstrapping was used to test the mediating effect of work engagement. If the confidence interval does not contain 0, the mediating effect is significant; otherwise, it is not significant. As shown in Table 4, the results of Model 1 and Model 2 show that morality and talent have a significant effect on individual creativity, and their indirect effect on individual creativity through work engagement is significant. The results of Model 3 show that the square term of morality has no significant direct effect on individual creativity but has a significant indirect effect on individual creativity through work engagement. The results of Model 4 show that the product term of morality and talent has a significant direct effect on individual creativity and a significant indirect effect on individual creativity through work engagement. The results of Model 5 show that the square term of talent has a significant direct effect on individual creativity and a significant indirect effect on individual creativity through work engagement. In conclusion, work engagement plays a mediating role in the U-shaped curve of morality and talent on individual creativity. Hypothesis 3 is supported.

**TABLE 4:** Mediating effect of work engagement.

		Individual creativity				
		Model 1	Model 2	Model 3	Model 4	Model 5
Intercept		2.333[1.837, 2.830]	2.105[1.657, 2.553]	1.495[1.071, 1.918]	1.315[0.908, 1.722]	1.422[1.018, 1.826]
Gender		-0.164[-0.272, -0.056]	-0.148[-0.256, -0.039]	-0.155[-0.265, -0.045]	-0.158[-0.265, -0.051]	-0.163[-0.272, -0.055]
Age		-0.012[-0.093, 0.069]	-0.018[-.100, 0.063]	-0.022[-0.105, 0.060]	-0.021[-0.102, 0.059]	-0.021[-0.102, 0.061]
Education level		0.103[0.030, 0.175]	0.094[0.021, 0.168]	0.112[0.038, 0.187]	0.128[0.055, 0.201]	0.114[0.041, 0.187]
Direct effect	Morality, β1	0.211[0.109, 0.312]				
	Talent, β2		0.165[0.076, 0.255]			
	Morality^2^, β3			0.047[0.000, 0.094]		
	Morality × Talent, β4				0.130[0.074, 0.186]	
	Talent^2^, β5					0.094[0.043, 0.146]
	Work engagement	0.426[0.319, 0.533]	0.484[0.393, 0.576]	0.627[0.553, 0.702]	0.660[0.588, 0.732]	0.641[0.569, 0.712]
Indirect effect		0.314[0.233, 0.409]	0.319[0.235, 0.414]	-.156[-.202, -.089]	-0.191[-0.256, -0.088]	-.148[-0.232, -0.079]
R-sq		0.469***	0.464***	0.451***	0.474***	0.464***
F		65.720	64.497	61.092	67.141	64.375

*** indicates significance at the level of p < 0.001.

## Discussion

### Conclusion

In this study, polynomial regression and response surface analysis were used to explore the curvilinear effect of morality and ability fit on individual creativity and the mediating effect of work engagement in morality and ability matching relationships on individual creativity. The results showed the following. (1) Morality and talent interact to influence work engagement and individual creativity. With a combination of high morality and high ability, employees have the highest level of work engagement and individual creativity. (2) In the state of morality and ability fit, employees with high morality and ability are more creative than those with low morality and ability. (3) When there is a misfit of morality and ability, employees with high morality and low talent are more creative than those with low morality and high talent. (4) Work engagement mediates the effect of morality and talent fit on individual creativity.

### Theoretical Implications

This research contributes to the current research in the following aspects. First, by referring to the theory of person-environment fit, a theoretical model of the influence of morality-talent fit on individual creativity is established. The influence of different states of morality and talent fit and morality and talent misfit on individual creativity is compared, and the influence mechanism of the interaction effect of person-environment fit on individual creativity is clarified. The data obtained through a questionnaire survey verify the reliability of the theoretical model, expand research on the antecedent variable of creativity, and enrich the theory of person-environment fit.

Second, this study compared and analyzed the influence of morality-ability fit on the curve of work engagement, which enriches research on the influencing factors of work engagement. In the past, research on work engagement mainly considered the aspects of leadership style, job characteristics, and employee characteristics but did not consider the perspective of employees’ personal morality and ability fit.

Third, this paper discussed the mechanism of morality and talent fit in the process of person-environment fit on individual creativity and revealed the mediating role of work engagement in this process, providing references for further understanding the relationships among person-environment fit, work engagement and individual creativity.

### Practical Implications

A diverse workforce in organizational management is an important prerequisite for ensuring organizational vitality. This study emphasizes the importance of person-environment fit in the management of work engagement and creativity in the service industry organizations under the digital background and provides management ideas for managers to realize digital transformation of enterprises.

First, morality is more important than talent, and loyalty is more important than ability. When hiring, it is necessary to evaluate the match between the candidate and the organization’s values. At the same time, the organization should distinguish between those who are available and those who must be eliminated. Behind any phenomenon in an organization is the reflection of the way employees think and behave. A company needs to describe what it absolutely cannot tolerate from a cultural perspective. Based on certain principles, the enterprise should define its own cultural standards. If an employee violates these standards and does something the enterprise cannot tolerate, the employee will be eliminated.

Second, it is necessary to enrich the content of incentives to meet diversified demands. In the service enterprises in the process of digital transformation, there are more scientific and technological talents and knowledge talented employees, who want to perform their duties without fear of compromising their individuality, self-worth, and self-esteem. They usually perform better in a supportive, nonthreatening and enjoyable work environment. So the organization needs to provide employees with opportunities to freely express their opinions, establish a trust mechanism among employees, and create an atmosphere in which employees can freely share their emotions and attitudes to promote a high level of devotion to work among employees. In addition, employees should be allowed to participate in decision-making, granted freedom of work and independent decision-making rights, motivated to contribute their talents, and given opportunities to give full play to their values.

Third, it is necessary to provide learning opportunities to improve the ability of employees. Industry digitization takes data as the key element, and under the guidance and support of the new generation of digital technology, digitization upgrading, transformation and reconstruction are carried out for the total elements of the upstream and downstream of the industrial chain. Learning ability of organizations is the base of employee learning, which could promote the growth of employees’ knowledge, skills and attributes. When employees feel that their organization is making a serious effort to improve their capabilities, they will demonstrate a high level of work engagement and individual creativity. Therefore, the organization should establish and share a list of capabilities with all departments to better promote the training of cross-industry and integrated talents who are proficient in supply demand coordination and consumption pattern innovation in the service industry, as well as in digital technology.

### Limitations and Discussion

Like any other study, this research also has limitations. First, the sample data are derived from cross-sectional data with no time difference. Furthermore, the measurement of the same subjects by self-reported survey data may have common method deviations. Second, the research is based on samples collected by China’s service industry groups and was not extended to different sample groups for comparative study. Third, the quadratic response surface regression method is an indirect measurement strategy that is the result of a cognitive comparison between individual and environment perception. This process is based on the evaluation of subjective comparison and ignores the self-evaluation of the person and environment fit in the comparison process. The impact of these factors will be taken into account in the future for further exploration and improvement.

## Data Availability Statement

The original contributions presented in the study are included in the article/supplementary material, further inquiries can be directed to the corresponding author/s.

## Author Contributions

DJ was responsible for the design of the research. ZC was responsible for the collection of data. SW was responsible for the derivation of theory and hypothesis. TL was responsible for the analysis and collation of data. HZ and QC were responsible for the organization and communication. All authors contributed to the article and approved the submitted version.

## Conflict of Interest

The authors declare that the research was conducted in the absence of any commercial or financial relationships that could be construed as a potential conflict of interest.

## Publisher’s Note

All claims expressed in this article are solely those of the authors and do not necessarily represent those of their affiliated organizations, or those of the publisher, the editors and the reviewers. Any product that may be evaluated in this article, or claim that may be made by its manufacturer, is not guaranteed or endorsed by the publisher.
